# Primary tumor resection with or without metastasectomy for left- and right-sided stage IV colorectal cancer: an instrumental variable analysis

**DOI:** 10.1186/s12876-022-02184-2

**Published:** 2022-03-09

**Authors:** Yi-Chen Yao, Jun-Quan Chen, Ling Yin, Wu-Hao Lin, Jian-Hong Peng, Wen-Hua Fan

**Affiliations:** 1 Department of Medical Oncology, State Key Laboratory of Oncology in South China, Collaborative Innovation Center of Cancer Medicine, Sun Yat-Sen University Cancer Center, Guangzhou, 510060 People’s Republic of China; 2 Department of Clinical Research, State Key Laboratory of Oncology in South China, Collaborative Innovation Center for Cancer Medicine, Sun Yat-Sen University Cancer Center, Guangzhou, 510060 People’s Republic of China; 3 Department of Colorectal Surgery, State Key Laboratory of Oncology in South China, Collaborative Innovation Center for Cancer Medicine, Sun Yat-Sen University Cancer Center, 651 Dong Feng Road East, Guangzhou, 510060 People’s Republic of China

**Keywords:** Colorectal cancer, Primary tumor resection, Metastasectomy, Instrumental variable analysis, SEER

## Abstract

**Background:**

Whether primary tumor location (PTL) is predictive of survival benefits following primary tumor resection plus metastasectomy (PMTR) and primary tumor resection (PTR) alone in stage IV colorectal cancer patients is not known. We sought to address this issue by employing instrumental variable analysis to evaluate the efficacy of PMTR and PTR with stratification for primary tumor location in stage IV colorectal cancer patients.

**Patients and methods:**

Stage IV colorectal cancer patients diagnosed between January 1, 2005 and December 31, 2015 were identified from the Surveillance, Epidemiology, and End Results Program of the National Cancer Institute. To account for both measured and unmeasured confounders, the efficacy of PMTR and PTR in the left- and right-sided subgroups was evaluated using instrumental variable analysis, with the health service area as the instrument variable. Overall survival (OS) was the primary outcome of interest.

**Results:**

A total of 50,333 eligible patients were analyzed (left-sided, n = 29,402 and right-sided, n = 20,931). OS was significantly better with PMTR than with other treatments (PTR, metastasectomy only, or no surgery) in patients with left-sided tumors (hazard ratio [HR] = 0.37 [95% CI 0.24–0.58], *P* < 0.001), but not in patients with right-sided tumors (HR = 0.98 [95% CI 0.65–1.47], *P* = 0.910; interaction test *P* < 0.001). OS was comparable in patients treated with PTR and those treated with no surgery in both the left-sided (HR = 1.11 [95% CI 0.68–1.81], *P* = 0.690) and right-sided (HR = 0.85 [95% CI 0.50–1.43], *P* = 0.530; interaction test *P* = 0.466) subgroups.

**Conclusions:**

PMTR appears to only benefit patients with left-sided stage IV colorectal cancer but not those with right-sided tumors. PTR does not improve OS, regardless of primary tumor location. When selecting patients for PMTR, primary tumor location should be considered. Overuse of PTR should be avoided.

**Supplementary Information:**

The online version contains supplementary material available at 10.1186/s12876-022-02184-2.

## Highlights

Increased use of primary tumor resection plus metastasectomy benefited patients with left-sided tumors but not those with right-sided tumors.

Increased use of primary tumor resection alone did not improve survival in either left or right-sided tumor group.

Overuse of palliative primary tumor resection should be avoided.

**Supplementary Information:**

The online version contains supplementary material available at 10.1186/s12876-022-02184-2.

## Introduction

According to GLOBOCAN, colorectal cancer (CRC) accounted for 10% of all cancers and for 9% of all cancer deaths in 185 countries in 2018 [1]. Approximately 20% of CRC patients have distant metastases at the time of diagnosis [2]. In those with resectable primary tumor and synchronous metastases, staged or simultaneous primary tumor resection plus metastasectomy (PMTR) leading to no evidence of disease (NED) can improve long-term survival [3]. Previous study found metastasectomy would improve OS in left-sided colon cancer but not in right-sided colon cancer [4]. However, there is still a lack of consensus on the resectability of metastatic lesions, and previous population-based studies have reported a potential underutilization of PMTR among stage IV CRC patients [5, 6]. According to the National Comprehensive Cancer Network (NCCN) Clinical Practice Guidelines, patients with symptomatic primary tumor (i.e., with obstruction, bleeding, and so on) should receive primary tumor resection (PTR), but those with asymptomatic metastatic CRC (stage IV CRC) should receive systemic chemotherapy as the initial treatment as it is still uncertain whether PTR improves outcomes in this group [7–13]. Recently, a randomized trial has demonstrated PTR is not associated with survival benefit in asymptomatic patients [14]. Although there is a decline in the number of PTRs being performed, it continues to be overused in clinical practice [15].

Depending on the differences in embryonic origin, the colon had divided into right-sided and left-sided. Right-sided CRC occurs in cecum, ascending colon, hepatic flexure and transverse colon, while left-sided CRC occurs in splenic flexure, descending colon, sigmoid colon and rectal. Accumulating evidence has revealed that tumor arising from different sides of the colon exhibit different features, such as epidemiological incidence, physiological characteristics, molecular alterations, and even survival outcome [16–18]. PTL was found to be a independent prognostic factor in stage IV CRC patients treated with systemic therapy and patients with left-sided tumors had a significantly better prognosis than those with right-sided tumors [19]. Price et al. study have shown that overall survival (OS) of stage IV CRC receiving hepatic surgery with or without chemotherapy had no significant difference by site, but OS of patients receiving hepatic chemotherapy only had markedly improved in patients with left-sided than those with right-sided [20]. So far, however, most of PTR or PMTR of stage IV CRC studies were based on all CRC populations, there have been no studies examining how PTL affects the outcomes of PMTR or PTR. The survival benefit of PMTR and PTR for site-specific stage IV CRC remains unclear.

Instrumental variable (IV) analysis, first introduced by Brookhart from econometrics to observational study [21], is a method useful in controlling both the measured and potential unmeasured confounders and to estimate causal-effect of treatment by relying on natural variation in treatment choice. In this study, we employed IV analysis to determine whether PTL affects survival outcomes of PMTR or PTR in stage IV CRC patients.

## Methods

### Patient eligibility and exclusion criteria

This population-based study used data extracted from the November 2018 ASCI text–data version of the Surveillance, Epidemiology, and End Results (SEER) Program of the National Cancer Institute, which covers ~ 28% of all cancer cases in the US [22]. We identified patients with CRC using the International Classification of Diseases for Oncology (ICD-O-3) site codes C180, C182 to C189, C199, and C209. We only considered patients with adenocarcinoma identified by the ICD-O-3 histology codes 8140, 8144, 8210, 8211, 8220, 8221, 8261, 8262, 8263, 8480, and 8481. Stage IV disease was identified based on the North American Association of Central Cancer Registries (NAACCR) items 790, 810, and 2980. We only included patients diagnosed with CRC in and after year 2005—i.e., after the two landmark targeted therapeutic agents cetuximab and bevacizumab were approved for use in metastatic CRC by the US Food and Drug Administration [23]. We excluded patients who (1) were < 18 years old; (2) did not have histologically confirmed diagnosis; (3) had previous history of other primary malignancy; (4) had been diagnosed only after death (e.g., at autopsy); (5) had incomplete follow-up information; or (6) had incomplete information on surgery to the primary or metastatic sites.

### Study endpoint and the examined demographic and clinical variables

OS—defined as the time from diagnosis to the date of death due to any cause—was the primary outcome of interest. The examined covariates included race, age, sex, marital status, year of diagnosis, health service area (HSA), tumor grade, tumor location, tumor size, American Joint Committee on Cancer (AJCC) T and N category, surgery to the primary site, and surgery to the metastatic site. Following the criteria used in prior studies, patients with cancer of the cecum, ascending colon, and transverse colon were defined as having right-sided tumors, and patients with cancer of the splenic flexure, descending colon, sigmoid colon, rectosigmoid junction, and rectum were considered as having left-sided tumors [24].

### Study design

This study is a population-based cohort study extracted from the Surveillance, Epidemiology, and End Results Program of the National Cancer Institute with stage IV colorectal cancer patients diagnosed between January 1, 2005 and December 31, 2015. To account for both measured and unmeasured confounders, the efficacy of PMTR and PTR in the left- and right-sided subgroups was evaluated using instrumental variable analysis, with the health service area as the instrument variable. Overall survival (OS) was the primary outcome of interest. Individual consent for this retrospective analysis was waived.

### Statistical analysis

We applied instrumental variable analytical methods, which can account for both measured and unmeasured confounders. Most of observational studies cannot achieve the inference of causality because the limitations and bias of design of studies (especially the unmeasured confounders). The unmeasured confounders are associated to exposure and outcome, which are important to drawing causality in observational studies [25–28].

IV requires one or more instruments, which are associated with exposure but are not associated with other confounding factors, and has no directly relationship with outcome variable. Two-stage predictor substitution (2SPS) and two-stage residual inclusion (2SRI) are two commonly used IV approaches, which are used in nonlinear models. We chose 2RSI for the instrumental variable analysis rather than 2SPS because the former was more consistent and less biased for solving problem of nonlinear models endogeneity [29, 30].

In contrast to a randomized controlled trial that identifies the average treatment effect [31, 32], instrumental variable analysis estimates the treatment effect for the marginal patients, i.e., the patients in whom the likelihood of undergoing the treatment depends on the instrumental variable [33]. The HSA—defined as one or more counties that are relatively self-contained with respect to the provision of routine hospital care [34]—was adopted as the instrumental variable because the difference in the use of PTR and PMTR across different HSAs is more likely to be associated with nonmedical factors (e.g., local treatment practices) than with differences in cancer characteristics. Each HSA with fewer than 50 cases was combined with the nearest (in terms of geographic distance) HSA. We used the Durbin–Wu–Hausman test of endogeneity to decide on the necessity of performing instrumental variable analysis; a significant result indicated that standard multivariable regression was likely to provide biased results [31].

Instrumental variable analysis was performed separately in the left-sided and right-sided tumor subgroups. Since race and the proportions of patients receiving PTR and PMTR may vary simultaneously across HSAs, we also performed stratified analysis. Taking into account the influence of medical resources and other factors, the PTR rate may be higher in areas with high PMTR rate. To evaluate the treatment effect of PMTR versus other strategies (i.e., PTR, metastasectomy-only, and no surgery to the primary or metastatic sites), we applied a 2SRI stratified by race (non-Hispanic white vs. non-Hispanic black vs. others) and the proportion of patients receiving PTR (below the median vs. equal to or above the median) [30]. In the first stage, we fitted a stratified logistic model including the HSA’s proportion of patients receiving PMTR (below median vs. equal to or above the median) to predict the receipt of PMTR versus other strategies, and calculated the residual, which was defined as the observed probability minus the predicted probability of receiving PMTR. The residual was then included in the second stage to evaluate the impact of PMTR versus other strategies on OS using a stratified Cox analysis adjusted by an individual frailty term [35]. We further evaluated the treatment effect of PTR versus no surgery to the primary or metastatic sites using a similar 2SRI instrumental variable analysis stratified by race and the proportion of patients receiving PMTR (below median vs. equal to or above the median).

Statistical significance was set as *P* < 0.05 in a two-tailed test. Statistical analysis was performed using R version 3.5.1 (http://www.r-project.org). The data cleaning and cleansing and model building relied on the R packages “*tidyr*”, “*dplyr*”, “*tibble*”, “*data.table*”, “*tableone*”, “*caret*”; survival analysis and Cox analysis, with or without IV correction relied on R packages “*rms*”, “*survival*”, and “*survminer*”; and figure drawing for using R package “*ggplot2*”.

## Results

### Patient characteristics

We identified 50,333 patients diagnosed with stage IV CRC between 2005 and 2015 (Additional file 1: Fig. S1).

Additional file 2: Table S1 summarizes the baseline characteristics of the patients in the different groups. Age, marital status, race, and tumor characteristics were imbalanced between PMTR and non-PMTR groups and similar results were also observed in the PTR versus non-PTR group.

Missing data for tumor characteristics (i.e., tumor size, tumor grade, T stage, and N stage) were more common in the non-PMTR group and the non-surgery group, suggesting these variables comprised a mixture of clinical/preoperative and postoperative pathologic information. Therefore, these variables were not qualified confounding factors for the survival impact of PMTR and PTR.

### Multivariable Cox analysis of survival impact of PMTR/PTR in left- and right-sided CRC subgroups

The fully-adjusted Cox models are shown in Additional file 3: Table S2.

In multivariate regression analysis, after adjusting for race, age, sex, marital status, and year of diagnosis, PMTR was found to be a predictor of longer OS in both left-sided (HR = 0.60 [95% CI 0.57–0.63]; *P* < 0.001; Additional file 4: Fig. S2A) and right-sided (HR = 0.52 [95% CI 0.50–0.55]; *P* < 0.001; Additional file 4: Fig. S2B) stage IV CRC patients. Significantly better OS was seen with PTR than with no surgery in patients with left-sided and right-sided tumors (Additional file 4: Fig. S2C and S2D).

However, Durbin–Wu–Hausman tests were statistically significant (*P* < 0.01 for all), suggesting that unmeasured confounders may have biased the results. Therefore, we performed instrumental variable analysis.

### Instrumental variable analysis of survival impact of PMTR in left- and right-sided CRC subgroups

The directed acyclic graph for the relationship between an instrumental variable (HSA), treatment (PTR or PMTR), observed and unobserved confounding factors and the outcome (OS) had shown in Additional file 5: Fig. S3.

**Fig. 1:**
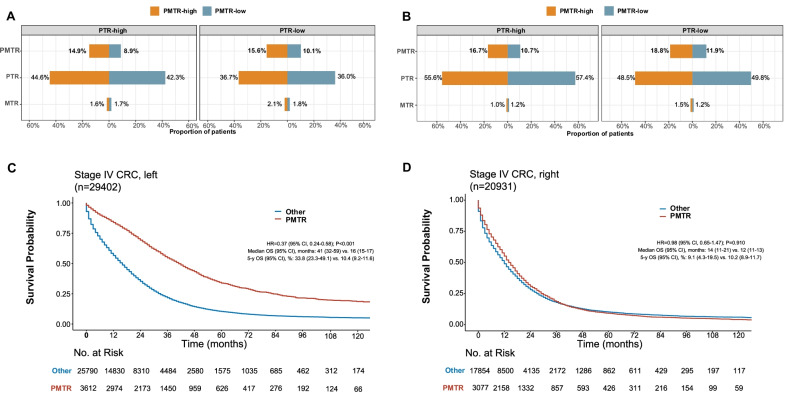
Proportions of patients receiving PMTR, PTR, or metastasectomy only, stratified by HSA PTR rate in the left-sided (**A**) and right-sided (**B**) CRC subgroups. Instrumental variable analysis–based OS for patients treated by PMTR or Other (i.e., PTR, metastasectomy only, or no surgery) in the left-sided (**C**) and right-sided (**D**) subgroups. The instrumental variable analysis was stratified by HSA PTR rate and race. Abbreviations: CRC, colorectal cancer; HR, hazard ratio; OS, overall survival; PMTR, primary tumor resection plus metastasectomy; PTR, primary tumor resection; MTR, metastasectomy

Figure 1A and B showed how surgical options differed between high-PMTR and low-PMTR HSAs.

In both the left-sided and right-sided subgroups, the proportion of patients receiving PTR or only metastasectomy was comparable between the high-PMTR region and low-PMTR region when stratified by PTR rate. We found significant non-arbitrary correlation between the HSA and the usage rate of PMTR in both the left-sided and the right-sided subgroups, as indicated by F statistics above the standard cutoff of 10 (271.5 and 229.5, respectively).

Table 1 shows the baseline characteristics of the left-sided and right-sided tumor groups in the high-PMTR region and low-PMTR region after stratification by HSA PTR rate.

**Table 1:** Comparison of baseline characteristics between the high-PMTR region and low-PMTR region, stratified by HSA PTR rate

	Left-sided, n = 29,402					Right-sided, n = 20,931				
	High PTR		Low PTR		*p*	High PTR		Low PTR		*p*
	High PMTR	Low PMTR	High PMTR	Low PMTR		High PMTR	Low PMTR	High PMTR	Low PMTR	
*Sex* = *Female*	1181 (38.7)	4910 (41.6)	4846 (41.7)	1219 (42.0)	0.017	1102 (50.6)	4220 (51.2)	4469 (52.7)	1027 (50.7)	0.1
*Age, years*					< 0.001					0.001
< 50	622 (20.4)	2283 (19.3)	2191 (18.8)	504 (17.3)		268 (12.3)	1006 (12.2)	998 (11.8)	210 (10.4)	
50–59	830 (27.2)	3104 (26.3)	2949 (25.3)	715 (24.6)		490 (22.5)	1631 (19.8)	1767 (20.8)	394 (19.5)	
60–69	812 (26.6)	3080 (26.1)	3046 (26.2)	738 (25.4)		597 (27.4)	2085 (25.3)	2099 (24.8)	528 (26.1)	
70–79	529 (17.3)	2031 (17.2)	2108 (18.1)	550 (18.9)		475 (21.8)	1912 (23.2)	1963 (23.1)	488 (24.1)	
≥ 80	261 (8.5)	1311 (11.1)	1340 (11.5)	398 (13.7)		350 (16.1)	1612 (19.5)	1653 (19.5)	405 (20.0)	
*Race*					< 0.001					< 0.001
Non-Hispanic White	2237 (73.2)	6486 (54.9)	8058 (69.3)	2096 (72.2)		1583 (72.6)	4820 (58.5)	5890 (69.5)	1506 (74.4)	
Non-Hispanic Black	401 (13.1)	1381 (11.7)	1779 (15.3)	179 (6.2)		379 (17.4)	1240 (15.0)	1719 (20.3)	168 (8.3)	
H ispanic	254 (8.3)	2267 (19.2)	647 (5.6)	465 (16.0)		145 (6.7)	1359 (16.5)	383 (4.5)	267 (13.2)	
Other	162 (5.3)	1675 (14.2)	1150 (9.9)	165 (5.7)		73 (3.3)	827 (10.0)	488 (5.8)	84 (4.1)	
*Marital status*					< 0.001					0.014
Widowed	343 (11.2)	1237 (10.5)	1424 (12.2)	388 (13.4)		376 (17.2)	1325 (16.1)	1510 (17.8)	331 (16.3)	
Married	1605 (52.6)	6155 (52.1)	5904 (50.7)	1504 (51.8)		1136 (52.1)	4225 (51.2)	4257 (50.2)	1079 (53.3)	
Other	1106 (36.2)	4417 (37.4)	4306 (37.0)	1013 (34.9)		668 (30.6)	2696 (32.7)	2713 (32.0)	615 (30.4)	
*Year of diagnosis*					0.825					0.144
2005–2007	803 (26.3)	3144 (26.6)	3048 (26.2)	761 (26.2)		568 (26.1)	2287 (27.7)	2300 (27.1)	529 (26.1)	
2008–2010	798 (26.1)	3142 (26.6)	3059 (26.3)	796 (27.4)		580 (26.6)	2308 (28.0)	2295 (27.1)	572 (28.2)	
2011–2015	1453 (47.6)	5523 (46.8)	5527 (47.5)	1348 (46.4)		1032 (47.3)	3651 (44.3)	3885 (45.8)	924 (45.6)	
*Tumor size, cm*					< 0.001					< 0.001
< 2	69 (2.3)	248 (2.1)	226 (1.9)	66 (2.3)		41 (1.9)	151 (1.8)	137 (1.6)	42 (2.1)	
≥ 2, < 4	453 (14.8)	1719 (14.6)	1672 (14.4)	374 (12.9)		330 (15.1)	1217 (14.8)	1237 (14.6)	269 (13.3)	
≥ 4, < 6	852 (27.9)	3197 (27.1)	3078 (26.5)	707 (24.3)		621 (28.5)	2254 (27.3)	2311 (27.3)	523 (25.8)	
≥ 6	762 (25.0)	2829 (24.0)	2866 (24.6)	636 (21.9)		683 (31.3)	2601 (31.5)	2606 (30.7)	583 (28.8)	
Unknown	918 (30.1)	3816 (32.3)	3792 (32.6)	1122 (38.6)		505 (23.2)	2023 (24.5)	2189 (25.8)	608 (30.0)	
*Tumor grade*					< 0.001					< 0.001
Grade1/2	1946 (63.7)	7374 (62.4)	7215 (62.0)	1816 (62.5)		1164 (53.4)	4529 (54.9)	4513 (53.2)	1067 (52.7)	
Grade3/4	590 (19.3)	2347 (19.9)	2088 (17.9)	544 (18.7)		695 (31.9)	2448 (29.7)	2472 (29.2)	601 (29.7)	
Unknown	518 (17.0)	2088 (17.7)	2331 (20.0)	545 (18.8)		321 (14.7)	1269 (15.4)	1495 (17.6)	357 (17.6)	
*T stage*					< 0.001					< 0.001
Tis	5 (0.2)	21 (0.2)	21 (0.2)	3 (0.1)		2 (0.1)	13 (0.2)	11 (0.1)	7 (0.3)	
T1	247 (8.1)	1250 (10.6)	1039 (8.9)	305 (10.5)		151 (6.9)	591 (7.2)	542 (6.4)	154 (7.6)	
T2	70 (2.3)	271 (2.3)	300 (2.6)	60 (2.1)		31 (1.4)	152 (1.8)	158 (1.9)	33 (1.6)	
T3	1340 (43.9)	4632 (39.2)	4596 (39.5)	1107 (38.1)		843 (38.7)	3116 (37.8)	3111 (36.7)	712 (35.2)	
T4	759 (24.9)	2778 (23.5)	2871 (24.7)	661 (22.8)		794 (36.4)	2794 (33.9)	2940 (34.7)	680 (33.6)	
Unknown	633 (20.7)	2857 (24.2)	2807 (24.1)	769 (26.5)		359 (16.5)	1580 (19.2)	1718 (20.3)	439 (21.7)	
*N stage*					< 0.001					0.015
N0	952 (31.2)	3748 (31.7)	3652 (31.4)	907 (31.2)		539 (24.7)	2175 (26.4)	2119 (25.0)	537 (26.5)	
N1	896 (29.3)	3608 (30.6)	3682 (31.6)	914 (31.5)		622 (28.5)	2362 (28.6)	2540 (30.0)	581 (28.7)	
N2	841 (27.5)	2835 (24.0)	2839 (24.4)	618 (21.3)		809 (37.1)	2801 (34.0)	2914 (34.4)	662 (32.7)	
Unknown	365 (12.0)	1618 (13.7)	1461 (12.6)	466 (16.0)		210 (9.6)	908 (11.0)	907 (10.7)	245 (12.1)	

*P* < 0.05 indicates a significant difference between the groups

HSA, health service area; PMTR, primary tumor resection plus metastasectomy; PTR, primary tumor resection

The proportion of missing data for tumor-associated variables was higher in the low-PMTR region, irrespective of PTL, because of the reasons mentioned above. For the other variables (except race), the absolute differences between the high-PMTR and low-PMTR regions were numerically small. Taken together, these findings suggest that the HSA PMTR rate satisfies the major requirements for a valid instrument. We then conducted instrumental variable analyses stratified for race and HSA PTR rate to evaluate the impact of PMTR on survival.

In the left-sided stage IV CRC group, survival was significantly better in those treated with PMTR than in those not treated with PMTR: median OS, 41 months (95% CI 32–59) versus 16 months (95% CI 15–17); HR = 0.37; 95% CI 0.24–0.58 (*P* < 0.001; Fig. 1C). However, in right-sided stage IV CRC, PMTR provided no significant survival benefit (HR = 0.98 [95% CI 0.65–1.47]; *P* = 0.910; Fig. 1D; interaction test *P* < 0.001).

**Fig. 2:**
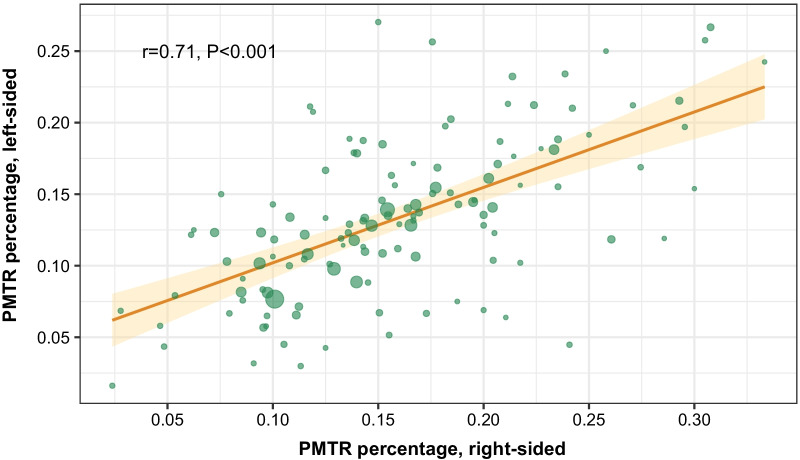
Correlation of PMTR rate in left-sided and right-sided tumors across different HSAs. The circle size is proportional to the number of patients in a given HSA. Abbreviations: PMTR, primary tumor resection plus metastasectomy

To determine whether the different results in the two PTL groups were due to different characteristics of the respective marginal populations for instrumental variable analyses, we assessed whether an HSA with a high PMTR rate for right-sided tumors also tended to have a high PMTR rate for left-sided tumors. The HSA-level PMTR rates for left-sided and right-sided tumors showed strong positive correlation (Pearson correlation coefficient weighted by HSA-level patient number, *r* = 0.71; *P* < 0.001; Fig. 2), suggesting that the PMTR practice pattern was similar for left-sided and right-sided tumors in each HSA and that the characteristics of the marginal populations in the left- and right-sided subgroups were similar in the instrumental variable analyses.

### Instrumental variable analysis of survival impact of PTR in left- and right-sided CRC subgroups

**Fig. 3:**
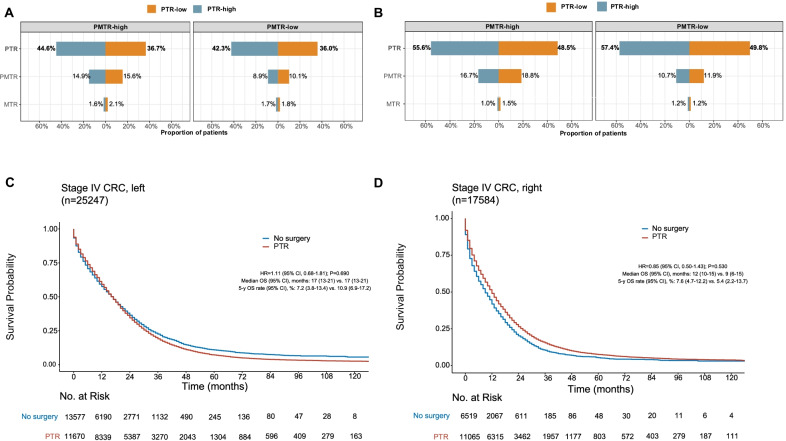
Proportion of patients receiving PTR, PMTR, or metastasectomy only, stratified by HSA PMTR rate in the left-sided (**A**) and right-sided (**B**) subgroups. Instrumental variable analysis–based OS for patients treated by PTR or No surgery in the left-sided (**C**) and right-sided (**D**) subgroups. The instrumental variable analysis was stratified by HSA PMTR rate and race. Abbreviations: CRC, colorectal cancer; HR, hazard ratio; OS, overall survival; PMTR, primary tumor resection plus metastasectomy; PTR, primary tumor resection; MTR, metastasectomy

Figure 3A and B showed the differences in surgical options between the high-PTR and low-PTR regions.

The proportions of patients receiving PMTR or only metastasectomy were similar between high-PTR regions and low-PTR regions after stratification by PMTR rate. Among patients treated with PTR or no surgery, we found a robust association between HSA and use of PTR in both left-sided and right-sided CRC subgroups (F statistics of 58.2 and 58.3, respectively). Baseline covariates (except race) were also comparable between the high-PTR and low-PTR regions (Additional file 6: Table S3).

After stratification by race and HSA PMTR rate, instrumental variable analysis showed comparable survival with PTR and with no surgery among patients with left-sided CRC: median OS, 17 months (95% CI 13–21) versus 17 months (95% CI 13–21); HR = 1.11 (95% CI 0.68–1.81); *P* = 0.690 (Fig. 3C). Similarly, no significant difference was seen in the right-sided CRC group: median OS, 12 months (95% CI 10–15) versus 9 months (95% CI 6–15); HR = 0.85 (95% CI 0.50–1.43); *P* = 0.530; interaction test *P* = 0.466 (Fig. 3D). The correlation of PTR rate in left-sided and right-sided tumors across different HSAs was demonstrated in Additional file 7: Fig. S4.

## Discussion

To our knowledge, this is the first study to evaluate the causal impact of PMTR and PTR on OS in left- and right-sided stage IV CRC patients, using instrumental variable analysis to adjust for both measured and unmeasured confounders. We found significant survival benefit with PMTR only in left-sided stage IV CRC patients. It should be emphasized that this finding cannot be generalized to the entire right-sided stage IV CRC population, because results from instrumental variable analysis only pertain to the marginal patients whose choice of PMTR is directly influenced by regional practice patterns. Although it is not possible to accurately delineate the characteristics of this marginal population, it may comprise those in whom surgery cannot be performed for technical reasons although the lesions are potentially resectable. Based on the correlation between PMTR rates in left- and right-sided CRC patients (Fig. 2), we speculate that the surgery patterns in the left- and right-sided subgroups were similar across different HSAs, which suggests that the characteristics of the marginal populations in the two subgroups were similar in the instrumental variable analyses.

A possible explanation for the lack of benefit with PMTR in the right-sided subgroup is the more aggressive biological behavior of right-sided tumors. The levels of specific biomarkers, such as microsatellite instability (MSI), CpG island methylator phenotype (CIMP) level, *BRAF* and *KRAS* mutations, gradually increase from the distal to the proximal colon [18]. A previous study has found that right-sided advanced CRC is more likely to be associated with *RAS* and *BRAF* mutations [36]. It has been reported that *BRAF* V600E mutation and *KRAS* mutations are associated with a worse prognosis in stage IV CRC [37]. Herein, these biological distinctions between right- and left-sided colon cancers could also translate into important differences in survival. Patients with right-sided tumors have poorer response to chemotherapy and target agents like cetuximab, and also have shorter OS after curative-intent hepatectomy for liver metastasis [19, 38]. Based on our findings, PTL should be taken into consideration when deciding on the feasibility of PMTR, with more stringent selection criteria applied to patients with right-sided tumors. One strategy could be to select for PMTR only those who have shown good response to systemic therapy. Our findings also suggest that the overall treatment outcomes in the regions that underutilize PMTR could be improved through efforts to increase use of PMTR in the marginal population with left-sided tumors.

There is considerable controversy regarding whether or not an asymptomatic primary tumor should be removed in patients with incurable stage IV CRC. Our instrumental variable analysis shows that PTR is not associate with improved survival in either left- or right-sided stage IV CRC. The marginal patients may be probably those who were asymptomatic, among whom the PTR rate would vary greatly across different regions. However, it should be mentioned that several retrospective observational studies have reported definite survival benefits with PTR in stage IV CRC [8–10]. The non-concordance with our results could be explained by the differences in statistical methods and the selection bias that is likely in retrospective investigations. We used HSA as instrumental variable to control for both measured and unmeasured confounders that could lead to misinterpretation of the impact of PTR and to focus on the marginal (asymptomatic) patients. Moreover, we only included patients diagnosed between 2005 and 2015, when systemic chemotherapy and targeted therapy were well developed, whereas most of the previous studies included patients treated before 2006. Some previous studies, including a recent randomized study, have reported results similar to ours, that is, PTR does not provide any survival benefit over that provided with systemic chemotherapy [11, 13, 14]; our investigation further shows that this is true for both left- and right-sided stage IV CRC patients. According to previous US population-based studies, more than half of stage IV CRC patients have received palliative PTR even in recent time [8, 12], which highlights the need to avoid the overuse of PTR. Multidisciplinary conference before PTR may help avoid unnecessary surgery and the associated delay in start of chemotherapy or targeted therapy [39].

There are several limitations in our study. First, our results are valid only as far as the assumptions of the instrumental variable are satisfied. We have attempted to confirm that our instrumental variable met the requirements, but there may still be some unobserved instrumental variable-related factors that were not well balanced between the groups. For instance, we cannot exclude the possibility that the variation in resection rates across HSAs is related to the quality of operation in these hospitals. However, the balanced usage of other surgical options in high- and low-PMTR/PTR areas (Figs. 1A and 3A) suggested comparable surgical quality among these areas. Second, metachronous metastasis cases were not included in our study, and thus the survival impact of PMTR and PTR remains to be explored in this subgroup. Third, instrumental variable analysis estimates only the marginal effect on the population under study, and it is difficult to define who the marginal patients are; further investigation is necessary to delineate these patients so that patient selection for PMTR can be optimized. Other limitation is the absence of mutation status and not considering this in multivariate model as these are important prognostic factors. Finally, our findings need to be further validated in other independent cohorts.

## Conclusion

PMTR appears to provide survival benefit only for patients with left-sided stage IV CRC, while geographically increased use of PTR is not associated with improved OS regardless of PTL. Our results suggest that PTL should be taken into consideration when selecting patients for PMTR, and routine use of palliative PTR in asymptomatic patients should be avoided. Multidisciplinary conference may help in selection of the best surgical candidates. Further studies are required to identify the patients who could benefit from surgery.

## Supplementary Information

**Additional file 1: Fig. S1.** Study Flow Chart.

**Additional file 2: Table S1.** Patient Characteristics in the Different Groups. Abbreviations: PMTR, primary tumor resection plus metastasectomy; PTR, primary tumor resection.

**Additional file 3: Table S2.** Fully-adjusted Cox models. Abbreviations: HR, hazard ratio; CI, Confidence intervals.

**Additional file 4: Fig. S2.** Multivariable Cox Analysis Evaluating the Impact of PMTR and PTR on Overall Survival. Abbreviations: CRC, colorectal cancer; HR, hazard ratio; OS, overall survival; PMTR, primary tumor resection plus metastasectomy; PTR, primary tumor resection.

**Additional file 5: Fig. S3.** The directed acyclic graph.

**Additional file 6: Table S3.** Comparison of Baseline Characteristics Between the High-PTR Region and Low-PTR Region, Stratified by HSA PMTR Rate. Abbreviations: HSA, health service area; PMTR, primary tumor and metastasis resection; PTR, primary tumor resection.

**Additional file 7: Fig. S4.** Correlation of PTR Rate in Left-sided and Right-sided Tumors across Different HSAs. Abbreviations: PTR, primary tumor resection.

## Data Availability

The data that support the findings of this study are available from SEER database (https://seer.cancer.gov/data/). We have uploaded all code and data to github (https://github.com/chenjq1/medicine). Data sharing statement: Data are available in a public, open access repository.
