# Supplementary figures and images for: Primary tumor resection with or without metastasectomy for left- and right-sided stage IV colorectal cancer: an instrumental variable analysis
Source: BMC Gastroenterol. 2022 Mar 9;22:114. doi: 10.1186/s12876-022-02184-2 (PMC8908621; doi:10.1186/s12876-022-02184-2)

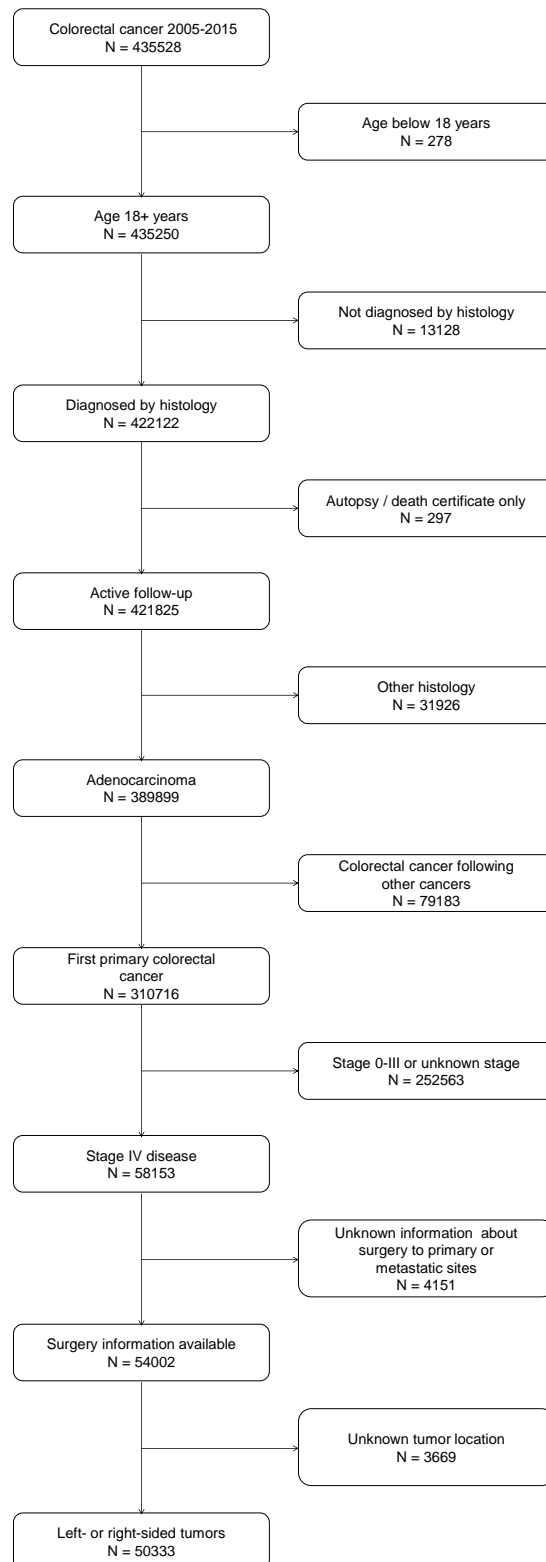

**eFigure 1. Study Flow Chart**

Supplement: Supplementary file 1 — Additional file 1: Fig. S1. Study Flow Chart. [file 12876_2022_2184_MOESM1_ESM.pdf]

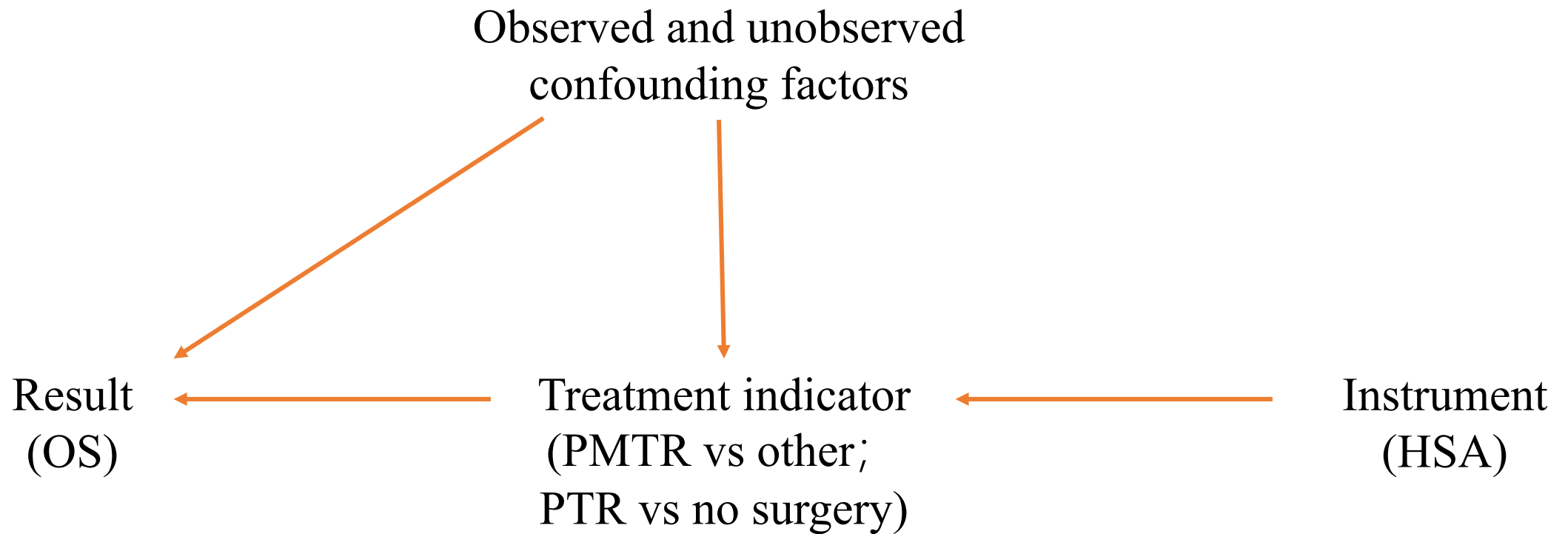

**eFigure 3. The directed acyclic graph**

Supplement: Supplementary file 5 — Additional file 5: Fig. S3. The directed acyclic graph. [file 12876_2022_2184_MOESM5_ESM.pdf]
