# Supplementary material for: Primary tumor resection with or without metastasectomy for left- and right-sided stage IV colorectal cancer: an instrumental variable analysis
Source: BMC Gastroenterol. 2022 Mar 9;22:114. doi: 10.1186/s12876-022-02184-2 (PMC8908621; doi:10.1186/s12876-022-02184-2)
Supplement: Supplementary file 2 — Additional file 2: Table S1. Patient Characteristics in the Different Groups. Abbreviations: PMTR, primary tumor resection plus metastasectomy; PTR, primary tumor resection. [file 12876_2022_2184_MOESM2_ESM.pdf]

Supplemental eTable 1. Patient Characteristics in the Different Groups

	Left-sided, n = 29402			Right-sided, n = 20931			Left-sided, n = 25247			Right-sided, n = 17584		
	**Other**	**PMTR**	**p**	**Other**	**PMTR**	**p**	**No Surgery**	**PTR**	**p**	**No Surgery**	**PTR**	**p**
**Number**	25790	3612		17854	3077		13577	11670		6519	11065	
**Sex = Female (%)**	10421 (40.4)	1735 (48.0)	<0.001	9051 (50.7)	1767 (57.4)	<0.001	5367 (39.5)	4806 (41.2)	0.008	3193 (49.0)	5713 (51.6)	0.001
**Age, years**			<0.001			<0.001			<0.001			<0.001
<50	4586 (17.8)	1014 (28.1)		1920 (10.8)	562 (18.3)		2195 (16.2)	2248 (19.3)		663 (10.2)	1211 (10.9)	
50-59	6593 (25.6)	1005 (27.8)		3525 (19.7)	757 (24.6)		3391 (25.0)	3035 (26.0)		1247 (19.1)	2214 (20.0)	
60-69	6815 (26.4)	861 (23.8)		4541 (25.4)	768 (25.0)		3598 (26.5)	3087 (26.5)		1626 (24.9)	2841 (25.7)	
70-79	4706 (18.2)	512 (14.2)		4203 (23.5)	635 (20.6)		2534 (18.7)	2103 (18.0)		1522 (23.3)	2634 (23.8)	
≥80	3090 (12.0)	220 (6.1)		3665 (20.5)	355 (11.5)		1859 (13.7)	1197 (10.3)		1461 (22.4)	2165 (19.6)	
**Race**			<0.001			<0.001			<0.001			<0.001
Non-Hispanic White	16421 (63.7)	2456 (68.0)		11647 (65.2)	2152 (69.9)		8498 (62.6)	7571 (64.9)		4156 (63.8)	7311 (66.1)	
Non-Hispanic Black	3325 (12.9)	415 (11.5)		3055 (17.1)	451 (14.7)		1856 (13.7)	1399 (12.0)		1232 (18.9)	1781 (16.1)	
Hispanic	3274 (12.7)	359 (9.9)		1891 (10.6)	263 (8.5)		1810 (13.3)	1396 (12.0)		705 (10.8)	1154 (10.4)	
Other	2770 (10.7)	382 (10.6)		1261 (7.1)	211 (6.9)		1413 (10.4)	1304 (11.2)		426 (6.5)	819 (7.4)	
**Marital status**			<0.001			<0.001			<0.001			<0.001
Widowed	3079 (11.9)	313 (8.7)		3130 (17.5)	412 (13.4)		1747 (12.9)	1289 (11.0)		1189 (18.2)	1910 (17.3)	
Married	13045 (50.6)	2123 (58.8)		8973 (50.3)	1724 (56.0)		6328 (46.6)	6429 (55.1)		3081 (47.3)	5746 (51.9)	
Other[a]	9666 (37.5)	1176 (32.6)		5751 (32.2)	941 (30.6)		5502 (40.5)	3952 (33.9)		2249 (34.5)	3409 (30.8)	
**Year of diagnosis**			0.004			0.044			<0.001			<0.001
2005-2007	6725 (26.1)	1031 (28.5)		4874 (27.3)	810 (26.3)		2854 (21.0)	3763 (32.2)		1427 (21.9)	3397 (30.7)	
2008-2010	6841 (26.5)	954 (26.4)		4852 (27.2)	903 (29.3)		3451 (25.4)	3247 (27.8)		1647 (25.3)	3132 (28.3)	
2011-2015	12224 (47.4)	1627 (45.0)		8128 (45.5)	1364 (44.3)		7272 (53.6)	4660 (39.9)		3445 (52.8)	4536 (41.0)	
**Tumor size, cm**			<0.001			<0.001			<0.001			<0.001

<2	491 (1.9)	118 (3.3)		301 (1.7)	70 (2.3)		200 (1.5)	276 (2.4)		81 (1.2)	212 (1.9)	
≥2, <4	3376 (13.1)	842 (23.3)		2450 (13.7)	603 (19.6)		897 (6.6)	2429 (20.8)		454 (7.0)	1973 (17.8)	
≥4, <6	6537 (25.3)	1297 (35.9)		4714 (26.4)	995 (32.3)		2049 (15.1)	4411 (37.8)		984 (15.1)	3709 (33.5)	
≥6	6087 (23.6)	1006 (27.9)		5368 (30.1)	1105 (35.9)		2509 (18.5)	3489 (29.9)		890 (13.7)	4440 (40.1)	
Unknown	9299 (36.1)	349 (9.7)		5021 (28.1)	304 (9.9)		7922 (58.3)	1065 (9.1)		4110 (63.0)	731 (6.6)	
**Tumor grade**			<0.001			<0.001			<0.001			<0.001
Grade 1/2	15717 (60.9)	2634 (72.9)		9370 (52.5)	1903 (61.8)		7139 (52.6)	8330 (71.4)		2601 (39.9)	6696 (60.5)	
Grade 3/4	4797 (18.6)	772 (21.4)		5194 (29.1)	1022 (33.2)		1902 (14.0)	2825 (24.2)		1179 (18.1)	3974 (35.9)	
Unknown	5276 (20.5)	206 (5.7)		3290 (18.4)	152 (4.9)		4536 (33.4)	515 (4.4)		2739 (42.0)	395 (3.6)	
**T stage**			<0.001			<0.001			<0.001			<0.001
Tis	49 (0.2)	1 (0.0)		31 (0.2)	2 (0.1)		40 (0.3)	4 (0.0)		21 (0.3)	6 (0.1)	
T1	2755 (10.7)	86 (2.4)		1402 (7.9)	36 (1.2)		2471 (18.2)	212 (1.8)		1270 (19.5)	103 (0.9)	
T2	581 (2.3)	120 (3.3)		294 (1.6)	80 (2.6)		213 (1.6)	353 (3.0)		30 (0.5)	261 (2.4)	
T3	9450 (36.6)	2225 (61.6)		6296 (35.3)	1486 (48.3)		2225 (16.4)	7130 (61.1)		484 (7.4)	5799 (52.4)	
T4	5978 (23.2)	1091 (30.2)		5794 (32.5)	1414 (46.0)		2112 (15.6)	3748 (32.1)		973 (14.9)	4755 (43.0)	
Unknown	6977 (27.1)	89 (2.5)		4037 (22.6)	59 (1.9)		6516 (48.0)	223 (1.9)		3741 (57.4)	141 (1.3)	
**N stage**			<0.001			<0.001			<0.001			<0.001
N0	8536 (33.1)	723 (20.0)		4696 (26.3)	674 (21.9)		5921 (43.6)	2401 (20.6)		2705 (41.5)	1863 (16.8)	
N1	7696 (29.8)	1404 (38.9)		5122 (28.7)	983 (31.9)		3424 (25.2)	4112 (35.2)		1669 (25.6)	3402 (30.7)	
N2	5699 (22.1)	1434 (39.7)		5812 (32.6)	1374 (44.7)		671 (4.9)	5001 (42.9)		129 (2.0)	5676 (51.3)	
Unknown	3859 (15.0)	51 (1.4)		2224 (12.5)	46 (1.5)		3561 (26.2)	156 (1.3)		2016 (30.9)	124 (1.1)	

[a]Includes patients treated with PTR, metastasectomy only, or no surgery.

P < 0.05 indicates a significant difference between the groups.

The “Other” group includes patients treated with PTR, metastasectomy only, or no surgery.

Abbreviations: PMTR, primary tumor resection plus metastasectomy; PTR, primary tumor resection.
