# Supplementary material for: Primary tumor resection with or without metastasectomy for left- and right-sided stage IV colorectal cancer: an instrumental variable analysis
Source: BMC Gastroenterol. 2022 Mar 9;22:114. doi: 10.1186/s12876-022-02184-2 (PMC8908621; doi:10.1186/s12876-022-02184-2)
Supplement: Supplementary file 3 — Additional file 3: Table S2. Fully-adjusted Cox models. Abbreviations: HR, hazard ratio; CI, Confidence intervals. [file 12876_2022_2184_MOESM3_ESM.pdf]

Supplemental eTable 2. Fully-adjusted Cox models

	Left-sided subgroup		Right-sided subgroup	
	**HR**	**95% CI%**	**HR**	**95% CI%**
**Treatment, reference: no surgery**				
PMTR	0.35	0.33-0.36	0.35	0.33-0.37
PTR-only	0.48	0.47-0.5	0.47	0.46-0.49
MTR-only	0.77	0.7-0.85	0.65	0.57-0.75
**Age, reference: <50**				
50-59	1.07	1.03-1.12	1.14	1.08-1.21
60-69	1.27	1.22-1.32	1.27	1.2-1.34
70-79	1.74	1.66-1.82	1.63	1.55-1.73
≥80	2.63	2.5-2.77	2.36	2.23-2.51
**Sex, reference: male**				
female	0.96	0.94-0.99	0.96	0.93-0.99
**Race, reference: Non-Hispanic white**				
Non-Hispanic black	1.21	1.16-1.26	1.07	1.03-1.11
Hispanic	0.95	0.91-0.99	0.92	0.87-0.97
Other	0.98	0.94-1.02	0.88	0.83-0.93
**Marital status, reference: Widowed**				
Married	0.84	0.8-0.88	0.85	0.81-0.89
Other	1.01	0.96-1.05	1.02	0.98-1.07
**Year of diagnosis, reference: 2005-2007**				
2008-2010	0.9	0.87-0.93	0.9	0.87-0.94
2011-2015	0.83	0.81-0.86	0.82	0.8-0.85

Abbreviations: HR, hazard ratio;CI, Confidence intervals
