# Supplementary material for: Primary tumor resection with or without metastasectomy for left- and right-sided stage IV colorectal cancer: an instrumental variable analysis
Source: BMC Gastroenterol. 2022 Mar 9;22:114. doi: 10.1186/s12876-022-02184-2 (PMC8908621; doi:10.1186/s12876-022-02184-2)
Supplement: Supplementary file 4 — Additional file 4: Fig. S2. Multivariable Cox Analysis Evaluating the Impact of PMTR and PTR on Overall Survival. Abbreviations: CRC, colorectal cancer; HR, hazard ratio; OS, overall survival; PMTR, primary tumor resection plus metastasectomy; PTR, primary tumor resection. [file 12876_2022_2184_MOESM4_ESM.pdf]

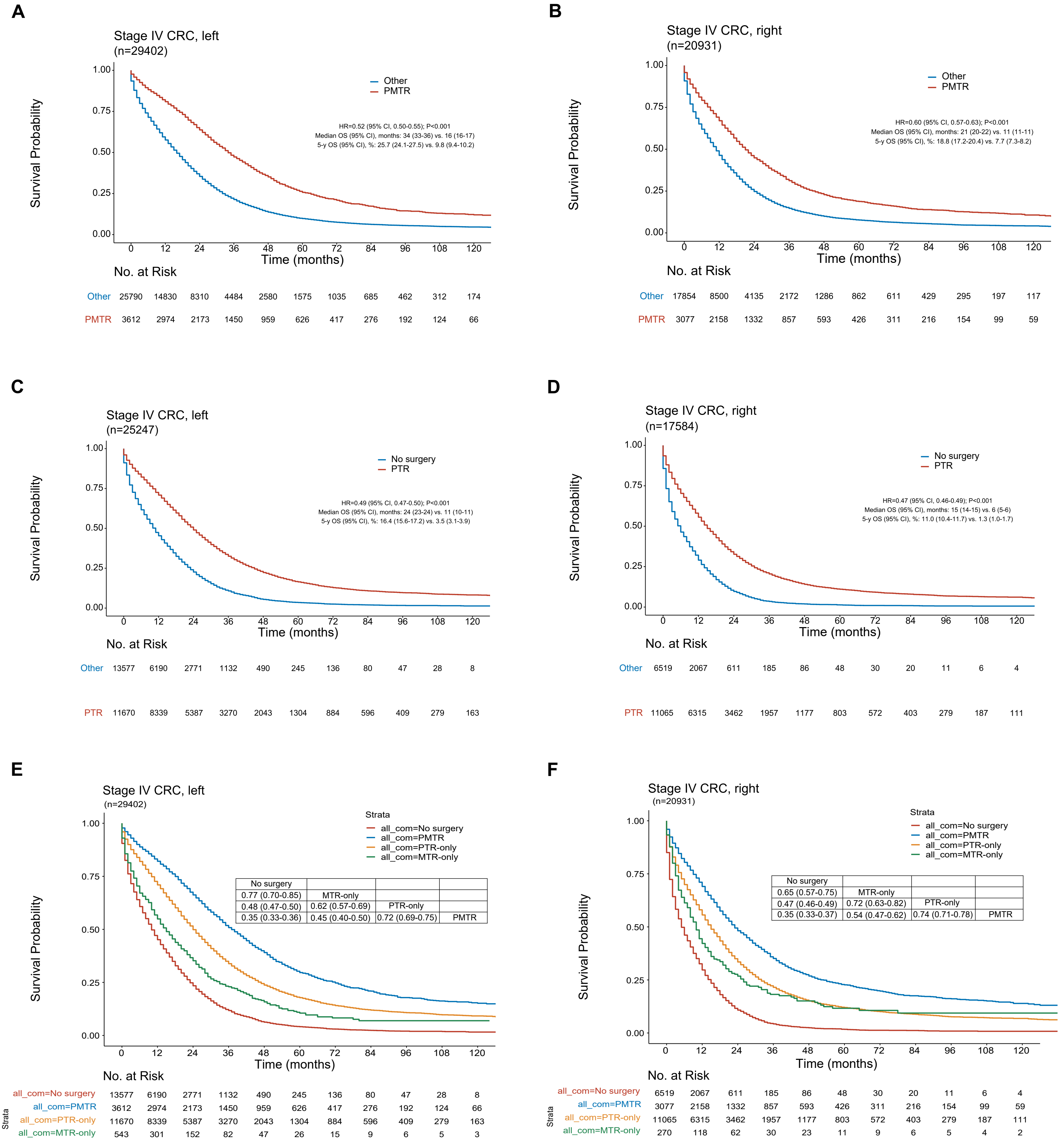


## eFigure 2. Multivariable Cox Analysis  Evaluating the Impact of PMTR and PTR on Overall Survival

(A) and (B): Multivariable Cox Analysis -based overall survival for patients treated by PMTR or Other (i.e., PTR, MTR, or no surgery) in the left-sided (A) and right-sided (B) subgroups.

(C) and (D): Multivariable Cox Analysis -based overall survival for patients treated by PTR or No surgery in the left-sided (C) and right-sided (D) subgroups.

(E) and (F): Multivariable Cox Analysis -based overall survival for patients treated by no surgery, PMTR, PTR-only or MTR-only in the left-sided (E) and right-sided (F) subgroups.

Abbreviations: CRC, colorectal cancer; HR, hazard ratio; OS, overall survival; PMTR, primary tumor resection plus metastasectomy; PTR, primary tumor resection.
