# Supplementary material for: Primary tumor resection with or without metastasectomy for left- and right-sided stage IV colorectal cancer: an instrumental variable analysis
Source: BMC Gastroenterol. 2022 Mar 9;22:114. doi: 10.1186/s12876-022-02184-2 (PMC8908621; doi:10.1186/s12876-022-02184-2)
Supplement: Supplementary file 6 — Additional file 6: Table S3. Comparison of Baseline Characteristics Between the High-PTR Region and Low-PTR Region, Stratified by HSA PMTR Rate. Abbreviations: HSA, health service area; PMTR, primary tumor and metastasis resection; PTR, primary tumor resection. [file 12876_2022_2184_MOESM6_ESM.pdf]

Supplemental eTable 3. Comparison of Baseline Characteristics Between the High-PTR Region and Low-PTR Region,Stratified by HSA PMTR Rate

	Left-sided mCRC patients, n=25247					Right-sided mCRC patients, n=17584				
	**High PMTR**		**Low PMTR**			**High PMTR**		**Low PMTR**		
	**High PTR**	**Low PTR**	**High PTR**	**Low PTR**	**p**	**High PTR**	**Low PTR**	**High PTR**	**Low PTR**	**p**
**SEX = Female**	940(36.9)	3906(40.8)	4276(40.5)	1051(41.1)	0.003	884(49.2)	3488(51.6)	3662(50.4)	872(49.5)	0.201
**Age, years**					<0.001					<0.001
<50	480(18.8)	1625(17.0)	1936(18.3)	402(15.7)		204(11.4)	688(10.2)	827(11.4)	155(8.8)	
50-59	683(26.8)	2368(24.7)	2734(25.9)	641(25.0)		394(21.9)	1340(19.8)	1394(19.2)	333(18.9)	
60-69	694(27.2)	2558(26.7)	2784(26.4)	649(25.4)		491(27.4)	1674(24.7)	1839(25.3)	463(26.3)	
70-79	456(17.9)	1820(19.0)	1866(17.7)	495(19.3)		403(22.5)	1607(23.8)	1707(23.5)	439(24.9)	
≥80	237(9.3)	1210(12.6)	1237(11.7)	372(14.5)		303(16.9)	1457(21.5)	1496(20.6)	370(21.0)	
**Race**					<0.001					<0.001
Non-Hispanic white	1855(72.7)	6620(69.1)	5755(54.5)	1839(71.9)		1299(72.4)	4647(68.7)	4209(58.0)	1312(74.5)	
Non-Hispanic black	346(13.6)	1485(15.5)	1263(12.0)	161(6.3)		318(17.7)	1426(21.1)	1129(15.5)	140(8.0)	
Hispanic	216(8.5)	536(5.6)	2040(19.3)	414(16.2)		126(7.0)	302(4.5)	1198(16.5)	233(13.2)	
Other	133(5.2)	940(9.8)	1499(14.2)	145(5.7)		52(2.9)	391(5.8)	727(10.0)	75(4.3)	
**Martial status**					<0.001					0.001
Widowed	294(11.5)	1237(12.9)	1153(10.9)	352(13.8)		315(17.5)	1284(19.0)	1210(16.7)	290(16.5)	
Married	1316(51.6)	4720(49.3)	5423(51.4)	1298(50.7)		916(51.0)	3304(48.8)	3665(50.5)	942(53.5)	
Other	940(36.9)	3624(37.8)	3981(37.7)	909(35.5)		564(31.4)	2178(32.2)	2388(32.9)	528(30.0)	
**Year of diagnosis**					0.785					0.187
2005-2007	674(26.4)	2491(26.0)	2779(26.3)	673(26.3)		462(25.7)	1862(27.5)	2032(28.0)	468(26.6)	
2008-2010	661(25.9)	2508(26.2)	2837(26.9)	692(27.0)		486(27.1)	1795(26.5)	2013(27.7)	485(27.6)	
2011-2015	1215(47.6)	4582(47.8)	4941(46.8)	1194(46.7)		847(47.2)	3109(46.0)	3218(44.3)	807(45.9)	
**Tumor size, cm**					<0.001					<0.001
<2	52(2.0)	162(1.7)	209(2.0)	53(2.1)		29(1.6)	105(1.6)	120(1.7)	39(2.2)	
≥2,<4	340(13.3)	1221(12.7)	1462(13.8)	303(11.8)		264(14.7)	904(13.4)	1044(14.4)	215(12.2)	
≥4,<6	683(26.8)	2394(25.0)	2778(26.3)	605(23.6)		498(27.7)	1753(25.9)	1991(27.4)	451(25.6)	
≥6	624(24.5)	2324(24.3)	2513(23.8)	537(21.0)		538(30.0)	2037(30.1)	2261(31.1)	494(28.1)	
Unknown	851(33.4)	3480(36.3)	3595(34.1)	1061(41.5)		466(26.0)	1967(29.1)	1847(25.4)	561(31.9)	
**Tumor grade**					<0.001					<0.001
Grade1/2	1592(62.4)	5786(60.4)	6520(61.8)	1571(61.4)		936(52.1)	3498(51.7)	3945(54.3)	918(52.2)	
Grade3/4	488(19.1)	1668(17.4)	2089(19.8)	482(18.8)		567(31.6)	1921(28.4)	2152(29.6)	513(29.1)	
Unknown	470(18.4)	2127(22.2)	1948(18.5)	506(19.8)		292(16.3)	1347(19.9)	1166(16.1)	329(18.7)	
**T stage**					<0.001					<0.001
Tis	5(0.2)	17(0.2)	19(0.2)	3(0.1)		1(0.1)	10(0.1)	9(0.1)	7(0.4)	
T1	224(8.8)	972(10.1)	1194(11.3)	293(11.4)		145(8.1)	506(7.5)	572(7.9)	150(8.5)	
T2	51(2.0)	229(2.4)	236(2.2)	50(2.0)		24(1.3)	122(1.8)	118(1.6)	27(1.5)	
T3	1047(41.1)	3454(36.1)	3940(37.3)	914(35.7)		663(36.9)	2330(34.4)	2685(37.0)	605(34.4)	
T4	617(24.2)	2254(23.5)	2432(23.0)	557(21.8)		622(34.7)	2176(32.2)	2380(32.8)	550(31.2)	
Unknown	606(23.8)	2655(27.7)	2736(25.9)	742(29.0)		340(18.9)	1622(24.0)	1499(20.6)	421(23.9)	
**N stage**					<0.001					0.108
N0	826(32.4)	3210(33.5)	3458(32.8)	828(32.4)		452(25.2)	1737(25.7)	1914(26.4)	465(26.4)	
N1	734(28.8)	2881(30.1)	3135(29.7)	786(30.7)		517(28.8)	2006(29.6)	2048(28.2)	500(28.4)	

N2	645(25.3)	2117(22.1)	2409(22.8)	501(19.6)	627(34.9)	2178(32.2)	2442(33.6)	558(31.7)
Unknown	345(13.5)	1373(14.3)	1555(14.7)	444(17.4)	199(11.1)	845(12.5)	859(11.8)	237(13.5)

P < 0.05 indicates a significant difference between the groups.

Abbreviations: HSA, health service area; PMTR, primary tumor and metastasis resection; PTR, primary tumor resection.
