# Supplementary material for: Primary tumor resection with or without metastasectomy for left- and right-sided stage IV colorectal cancer: an instrumental variable analysis
Source: BMC Gastroenterol. 2022 Mar 9;22:114. doi: 10.1186/s12876-022-02184-2 (PMC8908621; doi:10.1186/s12876-022-02184-2)
Supplement: Supplementary file 7 — Additional file 7: Fig. S4. Correlation of PTR Rate in Left-sided and Right-sided Tumors across Different HSAs. Abbreviations: PTR, primary tumor resection. [file 12876_2022_2184_MOESM7_ESM.pdf]

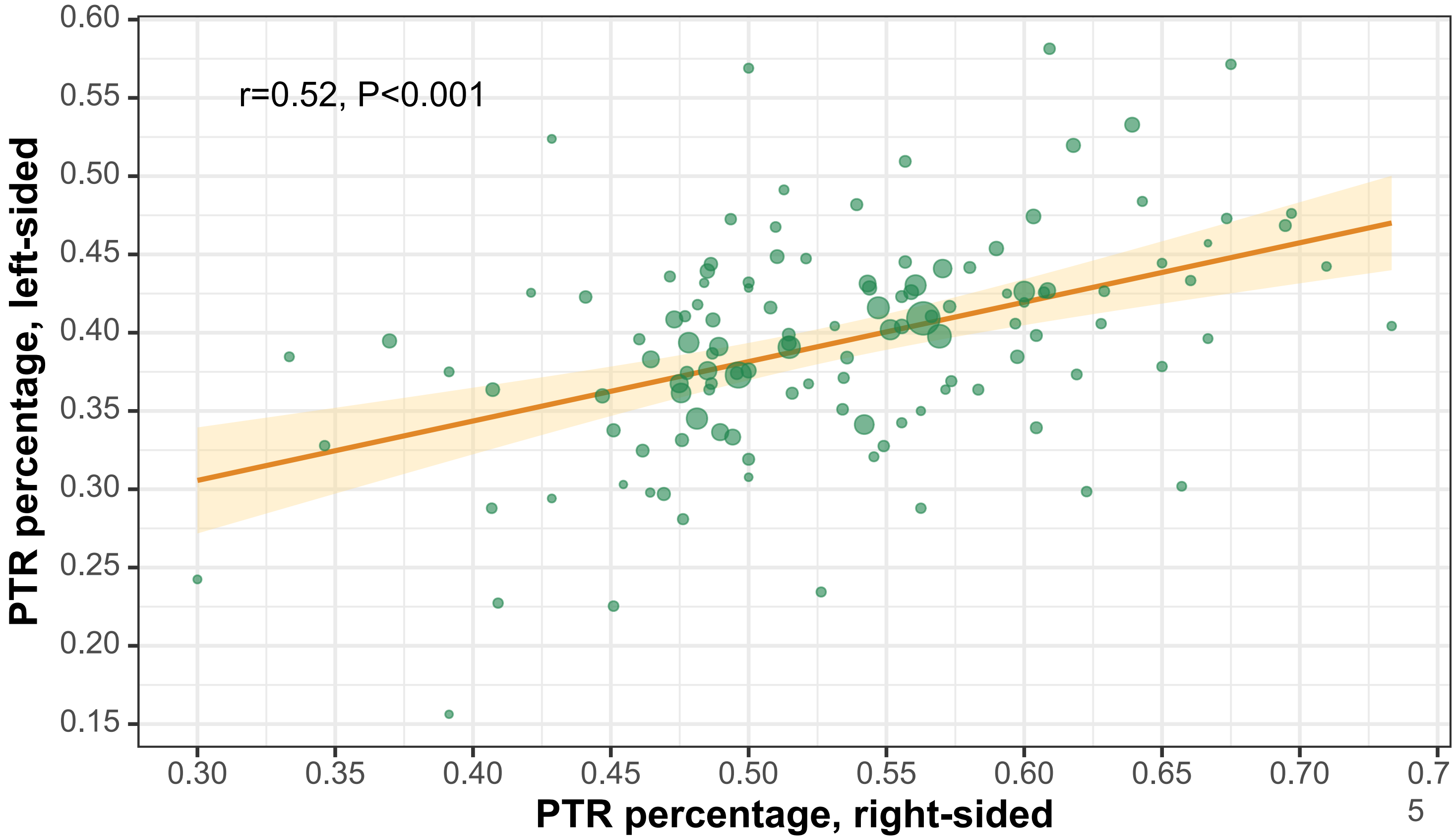

eFigure 4. Correlation of PTR Rate in Left-sided and Right-sided Tumors across Different HSAs

The circle size is proportional to the number of patients in a given HSA.

Abbreviations: PTR, primary tumor resection.
